# In this Issue

**Published:** 2012

## Overview: Stress and Alcohol Use Disorders Revisited

Drinking alcohol has the unique ability to both relieve stress and to be the cause of it, creating in a sense a double-edged sword. Understanding the link between alcohol drinking, stress, and alcohol is a critical area for ongoing investigation and ultimately may help in the prevention and treatment of alcohol use disorders. According to Dr. Anthenelli, an individual’s reactions to stress vary according to a number of factors, such as his or her genetic makeup, environment, life events, gender, age, and type and duration of stress. In this article, Dr. Anthenelli reviews some of these risk factors and introduces the articles included in this issue. (pp. 386–390)

## Stress and Alcohol: Epidemiologic Evidence

Although exposure to stress often can contribute to alcohol use and the risk of alcohol use disorders (AUDs), its specific impact depends on the type, timing during the life course, duration, and severity of the stress experienced. Drs. Katherine M. Keyes, Mark L. Hatzenbuehler, Bridget F. Grant, and Deborah S. Hasin summarize the epidemiologic evidence demonstrating that stress associated with general life stressors (e.g., divorce or job loss); fateful/catastrophic events, such as exposure to disaster and terrorism; childhood adversities, such as maltreatment; and chronic minority stress (e.g., based on race/ethnicity or sexual orientation) all can affect alcohol consumption and AUD risk. (pp. 391–400)

## Alcohol and Stress in the Military

Alcohol misuse is a problem among a significant minority of the U.S. military. Military-related traumatic stress seems to elevate risk for individuals to misuse alcohol. The co-occurrence of posttraumatic stress disorders seems to play a major explanatory role in the association between military stress and alcohol misuse. In this article, Drs. Jeremiah A. Schumm and Kathleen M. Chard discuss the prevalence of alcohol misuse within the military and how screening and intervention for alcohol use disorders, particularly following exposure to military-related trauma, is clearly needed, as are integrated treatments that address conjoined alcohol and posttraumatic stress disorder problems. (pp. 401–407)

## Childhood Trauma, Posttraumatic Stress Disorder, and Alcohol Dependence

Early-childhood trauma is strongly associated with developing mental health problems, including alcohol dependence, later in life. People with early-life trauma may use alcohol to help cope with trauma-related symptoms. This article by Drs. Kathleen T. Brady and Sudie E. Back reviews the prevalence of early-childhood trauma and its robust association with the development of alcohol use disorders and posttraumatic stress disorder. It also examines the potential biological mechanisms by which early adverse experiences can result in long-lasting changes in neurobiology underlying this vulnerability, as well as pharmacological and behavioral interventions. (pp. 408–413)

## Anxiety and Alcohol Use Disorders: Comorbidity and Treatment Considerations

Cumulative evidence from epidemiological and clinical studies over the past few decades has demonstrated that anxiety disorders and alcohol use disorders (AUDs) commonly co-occur. This comorbidity has a substantial clinical impact and is associated with substantial societal costs. As Drs. Joshua P. Smith and Carrie L. Randall explain, several models can explain the development of comorbid anxiety and AUDs, including the common-factor model, self-medication pathway, and substance-induced pathway. Furthermore, the two conditions may influence and maintain each other independent of the development pathway. The authors also discuss the implications of the comorbidity between anxiety and AUDs with respect to diagnosis and treatment of both conditions. (pp. 414–431)

## How Does Stress Lead to Risk of Alcohol Relapse?

Recent findings on differences in stress responsivity in alcohol-dependent versus nondependent social drinkers demonstrate alterations in stress pathways that may explain the significant contribution of stress-related mechanisms on craving and relapse susceptibility. This article by Dr. Rajita Sinha examines the stress-related processes that influence alcohol relapse, starting with an example of the stress-and alcohol cue-related experiences that increase alcohol-seeking and relapse susceptibility in an alcohol-dependent person. (pp. 432–440)

## Neural Pathways of Stress Integration: Relevance to Alcohol Abuse

Stress is a critical component in the development, maintenance, and reinstatement of addictive behaviors, including alcohol use. In this article, Dr. James P. Herman reviews the organization of neurocircuits that regulate stress responses, focusing on the hypothalamic–pituitary–adrenal (HPA) axis, which is of particular relevance to addictive processes. It also discusses areas of intersection between stress and reward pathways, as these are likely important in mediating the deleterious effects of stress on substance abuse and addiction. (pp. 441–447)

## Effects of Alcohol Dependence and Withdrawal on Stress Responsiveness and Alcohol Consumption

Chronic alcohol exposure and repeated withdrawal can profoundly disturb the function of the body’s neuroendocrine stress response system—the hypothalamic–pituitary–adrenocortical (HPA) axis. A hormone, corticotropin-releasing factor (CRF), which is produced and released from the hypothalamus and activates the pituitary in response to stress, plays a central role in the relationship between stress and alcohol dependence and withdrawal. Dr. Howard Becker describes how chronic alcohol exposure and withdrawal lead to changes in CRF activity both within the HPA axis and in other extrahypothalamic brain sites. Such changes may increase the risk for relapse to drinking and may play a role in escalating alcohol consumption in people who are alcohol dependent. (pp. 448–458)

## Clinical Laboratory Stressors Used to Study Alcohol–Stress Relationships

A comprehensive understanding of the relationship between stress and alcohol use is important for understanding the risks of developing alcohol problems and subsequent relapse. In this article by Drs. Suzanne Thomas, Amy K. Bacon, Rajita Sinha, Magdelena Uhart, and Bryon Adinoff, the authors discuss some of the most common physical, psychological, and pharmacological stressors used in stress-induction studies designed to reveal details about the relationship between stress reactivity and alcohol use and abuse. (pp. 459–467)

## Stress and the HPA Axis: Role of Glucocorticoids in Alcohol Dependence

Large epidemiological studies have reported that a variety of stressors are associated with increased alcohol consumption and binge drinking. As described by Drs. Mary Ann C. Stephens and Gary Wand, a hormonal response system to stress, the hypothalamic–pituitary–adrenal (HPA) axis, and in particular a stress hormone, cortisol, mediate this association. Alterations in HPA axis activity can play different roles at different stages of alcohol dependence. For example, cortisol can interact with the brain’s reward system, which may contribute to alcohol’s reinforcing effects, particularly during early stages of dependence. Cortisol also can influence a person’s cognitive processes, promoting habit-based learning, which may contribute to habit formation and risk of relapse. Finally, cortisol levels during abstinence may be useful clinical indicators of relapse vulnerability in alcohol-dependent people. (pp. 468–483)

## Genetic and Environmental Determinants of Stress Responding

The development of alcohol dependence is a complex process that is influenced by both genetic and environmental risk factors. For example, genetic variations in certain brain signaling pathways regulating reward, impulsivity and stress response influence a person’s liability for alcohol dependence. In this review, Dr. Toni-Kim Clarke, Ms. Charlotte Nymberg, and Dr. Gunter Schumann explore the contributions of some of the neurobiological systems that are important for the development of alcohol dependence, including the dopamine system and the body’s stress response systems—the hypothalamic–pituitary–adrenal axis and the corticotropin-releasing factor system. (pp. 484–494)

## Stress, Epigenetics, and Alcoholism

Stress and associated disorders (e.g., anxiety) are crucial factors in the development of and relapse to alcohol and drug dependence. One molecule that may help mediate the relationship between stress and alcohol consumption is brain-derived neurotrophic factor (BDNF). Mr. Sachin Moonat and Dr. Subhash C. Pandey describe how epigenetic mechanisms may influence BDNF activity and alter the relationships between stress, anxiety, and alcoholism. (pp. 495–505)

## Resilience to Meet the Challenge of Addiction: Psychobiology and Clinical Considerations

Acute and chronic stress play an important role in the development of addiction. In this article, Drs. Tanja N. Alim, William B. Lawson, Adriana Feder, Brian M. Iacoviello, Ms. Shireen Saxena, Mr. Christopher R. Bailey, Ms. Allison M. Greene, and Dr. Alexander Neumeister examine the interindividual stress responsivity at multiple phenotypic levels, ranging from psychological differences in the way people cope with stress to differences in neurochemical or neural circuitry function. The ultimate goal of such research is the development of strategies and interventions to enhance resilience and coping in the face of stress and prevent the onset of addiction problems or relapse. (pp. 506–515)

## Treatment of Alcohol Dependence With Drug Antagonists of the Stress Response

Studies have shown that alcohol-dependent people are more sensitive to relapse-provoking cues such as alcohol, negative affect, and stress. In addition, stress relief during protracted abstinence is thought to be a major motivation for excessive alcohol consumption. This article by Drs. Amanda E. Higley, George F. Koob, and Barbara J. Mason explores the relationship between chronic alcohol use, stress, and relapse that has implications for the treatment of alcohol dependence. (pp. 516–521)

